# The role of DNA topoisomerase 1α (AtTOP1α) in regulating arabidopsis meiotic recombination and chromosome segregation

**DOI:** 10.7717/peerj.17864

**Published:** 2024-08-28

**Authors:** Ibrahim Eid Elesawi, Ahmed M. Hashem, Li Yao, Mohamed Maher, Abdallah A. Hassanin, Diaa Abd El-Moneim, Fatmah A. Safhi, Nora M. Al Aboud, Salha Mesfer Alshamrani, Wael F. Shehata, Chen Chunli

**Affiliations:** 1 College of Life Science and Technology, Huazhong Agricultural University, Wuhan, China; 2 Agricultural Biochemistry Department, Faculty of Agriculture, Zagazig University, Zagazig, Egypt; 3 Biotechnology Department, Faculty of Agriculture, Al-Azhar University, Cairo, Egypt; 4 Genetics Department, Faculty of Agriculture, Zagazig University, Zagazig, Egypt; 5 Department of Plant Production, (Genetic Branch), Faculty of Environmental and Agricultural Sciences, Arish University, El-Arish, El-Arish, Egypt; 6 Department of Biology, College of Science, Princess Nourah bint Abdulrahman University, Riyadh, Saudi Arabia; 7 Department of Biology Faculty of Science, Umm Al‐Qura University, Makkah, Saudi Arabia; 8 Department of Biological Science, College of Science, University of Jeddah, Jeddah, Saudi Arabia; 9 College of Agriculture and Food Sciences, Department of Agricultural Biotechnology, King Faisal University, Al-Ahsa, Al-Ahsa, Saudi Arabia; 10 College of Environmental Agricultural Science, Plant Production Department, Arish University, Arish, North Sinai, Egypt; 11 National Key Laboratory for Germplasm Innovation and Utilization for Fruit and Vegetable Horticultural Crops, Huazhong Agricultural University, Wuhan, Hubei, China

**Keywords:** ATM, Centromere, DNA topoisomerase 1α, FISH, Meiosis, 45s rDNA

## Abstract

Meiosis is a critical process in sexual reproduction, and errors during this cell division can significantly impact fertility. Successful meiosis relies on the coordinated action of numerous genes involved in DNA replication, strand breaks, and subsequent rejoining. DNA topoisomerase enzymes play a vital role by regulating DNA topology, alleviating tension during replication and transcription. To elucidate the specific function of DNA topoisomerase 1α ( $AtTOP1 \alpha$) in male reproductive development of *Arabidopsis thaliana*, we investigated meiotic cell division in Arabidopsis flower buds. Combining cytological and biochemical techniques, we aimed to reveal the novel contribution of $AtTOP1 \alpha$ to meiosis. Our results demonstrate that the absence of $AtTOP1 \alpha$ leads to aberrant chromatin behavior during meiotic division. Specifically, the top1α1 mutant displayed altered heterochromatin distribution and clustered centromere signals at early meiotic stages. Additionally, this mutant exhibited disruptions in the distribution of 45s rDNA signals and a reduced frequency of chiasma formation during metaphase I, a crucial stage for genetic exchange. Furthermore, the atm-2×top1α1 double mutant displayed even more severe meiotic defects, including incomplete synapsis, DNA fragmentation, and the presence of polyads. These observations collectively suggest that $AtTOP1 \alpha$ plays a critical role in ensuring accurate meiotic progression, promoting homologous chromosome crossover formation, and potentially functioning in a shared DNA repair pathway with ATAXIA TELANGIECTASIA MUTATED (ATM) in Arabidopsis microspore mother cells.

## Introduction

Sexual reproduction is the dominant mode for most eukaryotic organisms, although the reasons behind this prevalence remain a subject of investigation (Ma & Shi, 2014; Plötner, 2023). In flowering plants, meiosis takes place within the anthers and ovules, a critical process for sexual reproduction in eukaryotes (Cui et al., 2024). Plants produce several flowers with readily detectable meiotic chromosomes, making them a valuable model for investigating the cellular processes that underpin meiosis (Prusicki et al., 2021). Meiosis facilitates the formation of haploid gametes through two meiotic divisions (meiosis I and II) after a single round of DNA replication (Zickler & Kleckner, 2023). This reduction in chromosome number from diploid (2n) to haploid (1n) is essential for the fusion of gametes during fertilization, restoring the diploid state in the offspring (Wang et al., 2021). The formation of a homologous chromosome connection, which ensures proper chromosome segregation during meiosis I, represents a critical step in prophase I. Synaptonemal complexes (SCs) and recombination nodules (RNs) are complexes associated with chromosome synapsis and recombination that can be seen in a variety of organisms (You et al., 2024). During prophase I of meiosis, homologous chromosomes undergo recombination, enabling the reciprocal exchange of genetic material between non-sister chromatids, thus becoming an essential source of genetic diversity (Hofstatter et al., 2021). During meiotic prophase I, programmed double-strand breaks (DSBs) occur in homologous chromosomes, catalyzed by SPO11 protein complexes (Ito & Shinohara, 2023). During meiosis, DSBs are processed to generate 3′ssDNA with a free 3′ hydroxyl end. These 3′ssDNA tails can initiate a DNA strand invasion step, where they anneal with homologous sequences on the partner chromosome. This homologous recombination process is essential for proper chromosome segregation during meiosis and enable crossover (Cos) or non-crossover repairs (Thangavel et al., 2023; Yamaya et al., 2023). Approximately 200 meiotic DSBs are produced in *Arabidopsis*, and only 10 are repaired as crossovers (Wang et al., 2021).

Following the occurrence of DNA breakage, SPO11 forms covalent bonds with the 5′ end of the DSBs; it’s important to note that SPO11 is subsequently removed before the repair of the DNA end. The RAD51 and DMC1 proteins are responsible for capturing and rebuilding nucleoprotein filaments, specifically targeting non-sister chromatids. Functional meiotic proteins for DNA repair and other factors mediate the second-end DSB capture, ligation, DNA synthesis, and CO-generating commitment (Ito & Shinohara, 2023).

Most organisms exhibit two distinct categories of crossover events (COs). Class I COs are characterized by their susceptibility to a phenomenon known as crossover interference. This phenomenon describes the reduced probability of additional COs occurring in close proximity to an existing CO. The ZMM proteins, specifically Zip1-3, Zip4/Spo22, and Msh4/Msh5, play a crucial role in mediating class I CO formation (Xin et al., 2021). Class II COs are non-interfering and rely on the Mus81 endonuclease (Desjardins et al., 2022). In addition to crossovers, meiotic DSB repair can utilize non-crossover processes like synthesis-dependent strand annealing (Lorenz & Mpaulo, 2022; Sen, Dodamani & Nambiar, 2023). In a subset of organisms, at least one crossover (CO) event per homologous chromosome pair is essential for accurate chromosome segregation during meiosis. However, a huge number of DSBs are repaired through non-CO mechanisms or even by interaction with sister chromatids (Lloyd, 2023; Wang et al., 2015).

Crossover recombinations create genetic variation as well as chiasmata formation. The chiasmata connect homologous chromosomes, thereby ensuring proper chromosome alignment and separation during meiosis (Kumar Koul & Nagpal, 2023). Therefore, it is necessary that crossover creation is closely controlled and that only a small number of DSBs are used to form mature crossovers (Alavattam et al., 2021). Recombination events are not randomly distributed, as in human, mouse, insect, yeast, and plant meiosis; moreover, crossovers close to centromeric and pericentromeric regions, and some telomeric regions, are suppressed, most probably to decrease the aneuploid threat (Ito & Shinohara, 2023; Kim & Choi, 2022).

Ataxia telangiectasia mutated (ATM) acts as a critical regulator in the DNA damage response pathway, promoting repair through phosphorylation of key proteins involved in various cellular processes, including cell cycle arrest checkpoints (Groelly et al., 2023; Herbst, Li & De Veylder, 2024). The protein kinase ATM acts as a critical regulator and sensor of DSBs during meiosis in various organisms. In Arabidopsis, ATM plays a pivotal role, impacting fertility and influencing numerous processes essential for successful meiotic completion. These processes include DSB formation and processing, DNA repair, the establishment of non-interference crossovers, and the assembly of the synaptonemal complex (SC) (Herbst, Li & De Veylder, 2024; Kurzbauer et al., 2021).

DNA topoisomerases play a critical role in developing appropriate DNA structure and topology, fixing kinks and torsions, and dealing with tight-twisted intermediates produced during replication and transcription. DNA topoisomerases influence DNA replication processes by reducing stress (Durand-Dubief et al., 2011; Gamarra & Narlikar, 2021; McKie, Neuman & Maxwell, 2021). In *Arabidopsis*, there are two topoisomerase type I genes, *TOP1α* and *TOP1β*, which are tandemly located on chromosome 5. The TOP1α and TOP1β proteins share 60% sequence similarity (Takahashi et al., 2002). The downregulation of *TOP1β* has no detectable effects; however, *TOP1α* knockout disrupts primordium initiation in shoot and floral meristem tissues, affecting plant phyllosphere guidance and overall plant architecture (Dinh et al., 2014; Zhang et al., 2022a). Furthermore, TOP1α functions in several developmental processes. For instance, photosynthesis-derived sugars promote TOP1α expression at the root tip to regulate TARGET OF RAPAMYCIN (TOR) expression and directly or indirectly maintain the quiescent center (QC), columella stem cell (CSC) identity and columella (COL) development (Zhang et al., 2022a); it is involved in determining seed size (Wang et al., 2024); it regulates Polycomb-group (PcG) proteins by changing nucleosome density in *Arabidopsis* (Liu et al., 2014; Yang et al., 2022); it influences floral transition by regulating the expression of *FLC* gene and close homologs; it regulates a central flowering repressor by influencing transcription machinery and histone modification (Gong et al., 2017; Zhong et al., 2019); and the inhibition of *TOP1α* results in defects in fertilization and spore formation in *Physcomitrium patens* (*P. patens*) while inhibition of *TOP1β* has no effect (Gu et al., 2022). TOP1α also emerges as a key regulator of RNA-DNA hybrids (R-loops) within the nucleus in response to stress signals. Deficiency in TOP1α function leads to accumulation of R-loops in plant nuclear chromatin (Kyung, Jeon & Lee, 2022; Shafiq et al., 2017). This research aims to study the role of TOP1α during meiosis in *Arabidopsis thaliana* and underscores its importance in meiotic recombination, fertility, and a decline in genetic crossing over.

## Materials and Methods

### Plant materials, crosses, and growth conditions

*A. thaliana* Columbia (Col-0) plants were used in this study. The *top1α1* had a T-DNA insertion in the eighth intron (Takahashi et al., 2002), and was obtained from the Taku Takahashi lab. The atm-2 mutant (SALK_006953) was obtained from the Salk Institute for Biological Studies. The double mutant was generated by crossing *top1α1* and *atm-2* mutants. Plants were maintained at ~22 °C and 60% humidity with light cycles of 16 h light/8 h dark.

### PCR genotyping, semi-quantitative PCR and qRT-PCR

Plant genotyping was performed using PCR with specific primers detailed in Table S1. These primers included top1α_LP, RP and LB; atm_LP, RP and SALK LBb1.3. All plant lines were in the Columbia (Col-0) genetic background. RNA extraction from seedlings was achieved using TRIzol reagent (Invitrogen, Waltham, MA, USA). Subsequently, first-strand cDNA synthesis was carried out from 3 μg of total RNA using 200 units (U) of M-MLV reverse transcriptase (Invitrogen, Waltham, MA, USA). qRT-PCR was performed using an optical 96-well plate in an ABI Step one plus PCR system (Applied Biosystems, Waltham, MA, USA) using SYBR Green Master Mix (Roche, Basel, Switzerland). *ACTIN8* served as the reference gene for the normalization of gene expression.

### Pollen grains, mature anther staining, and dissected tetrads

Pollen viability and mature anther phenotypes were assessed using Alexander stain according to the Gu method (Liu et al., 2023). Briefly, flower buds at stage 12 of anther development were fixed in 1 mL of Carnoy’s solution (6:3:1 ethanol:chloroform:acetic acid, v/v) for a minimum of 2 h. Following fixation, the Carnoy’s solution was removed, and the flower buds were dissected under a dissecting microscope. To differentiate viable from non-viable pollen grains, individual anthers were incubated with Alexander stain for 30 min at room temperature (25 °C). Subsequently, the anthers were mounted on a slide and visualized using an Olympus BX53F microscope (Olympus, Tokyo, Japan). This staining technique enabled the discrimination of viable pollen grains (magenta-red) from non-viable ones (blue or green) (Chang et al., 2014; Mao-Sen et al., 2021).

### Iodine pollen starch test

Pollen viability was assessed using a starch staining technique as described by Chang et al. (2021). Inflorescences were fixed and stored in 70% (*v*/*v*) ethanol. Flower buds were dissected under a dissecting microscope for anther isolation. Six anthers per flower were crushed on a microscope slide in a droplet of 1% (w/v) iodine-potassium iodide (I2/KI) solution. Excess solution was carefully withdrawn using forceps. The preparation was then observed under a light microscope at room temperature (25 °C) after coverslip placement.

### Separation and detection of tetrads

Inflorescences with unopened flowers were fixed with Carnoy’s fixative (3:1 100% ethanol, glacial acetic acid) overnight at 25 °C. Older buds outside the inflorescence containing obvious yellow anthers were removed, while sepals were left intact. The inflorescence was washed three times with water after preparation. Immature buds were dissected using a fine needle on a glass slide under a binocular microscope. Immature anthers were isolated from other floral organs in two drops of water. The anthers were then squeezed with sharp forceps to release the tetrads. The slide was air-dried, and tetrads were stained for 2–3 min with 0.01% basic fuchsine (1:3 3.0 g basic fuchsine in 100 mL 95% ethanol, 5% aqueous phenol). The slide was covered with a coverslip, and the red-stained tetrads were observed under a light microscope following previously recommended procedures (Prusicki et al., 2021).

### Chromosome distribution for meiotic chromosome detection with DAPI

The chromosome spread was determined by applying the protocol described previously (Prusicki et al., 2021; Wang et al., 2014). The inflorescences with unopened flowers were fixed with Carnoy’s solution at room temperature overnight. The buds rinsed three times every 10 min with 10 mM citrate solution. Then, old buds were removed containing yellow anthers that could be seen before adding the digestion-by-digestion cocktail (0.3% pectolyase, 0.3% cellulase, and 0.5% cytohelicase in 10 mM citrate buffer, pH 4.5) at 37 °C for 80 min. Then, we washed the set buds five times, every 5 min, with 10 mM citrate buffer, after stopping digestion on ice for 5 min. Following that, we placed a bud on the slide to dissect it, obtain anthers, and remove the rest of the flower. Anthers were mashed with sharp forceps in a drop of 60% acetic acid. Next, we moved the slide to a heat block, incubated it at 45 °C for 30 s, and added another drop for another 30 s. Then, 20 µL of freezing Carnoy’s fixative was added to a center sample to spread the meiotic chromosomes on the slide, which were left to air dry for approximately 5 min. We added 5 μL of DAPI to the slide, covered with a coverslip, and observed the chromosomes under a fluorescent microscope.

### Fluorescence *In Situ* Hybridization (FISH)

Chromosomes were separated according to Wang et al. (2014). Thereafter, 100 μL of 70% formamide was added to each dry slide dissolved in 2 × SSC buffer; then, the slide was covered with a parafilm piece and incubated in an oven at 80–90 °C for 5 min. The slides were dehydrated by treating with cold alcoholic dilutions at −20 °C (70%, 80%, 90%, and 100% ethanol) for 5 min for each dilution, and then left the slides to air dry. Following that, 10 μL of solution was mixed by combining 2 μL of the labeled probe (centromere or/and telomere or/and 45s rDNA probe, designed as shown in Table S2) with 8 μL the hybridization cocktail for each slide. We poured 10 μL mixture for each slide and covered with a coverslip; slides were incubated at 85 °C for 5 min to denature the mixture and then cooled directly on ice for 5 min. The slices were incubated in a slide box with high humidity to prevent the slices from drying out at 37 °C overnight while avoiding bright light. The covers were removed in a 2 × SSC buffer and then washed in 2 × SSC buffer three times for 15 min each time, and then one time with 1 × PBS buffer for 5 min. The slides were air-dried, 10 μL DAPI (4, 6-diamidino-2-phenylindole) was applied on each slide, and we covered with a coverslip. The slides were observed under a fluorescent microscope.

### Statistical analysis

All experiments were performed at least three times. For statistical comparisons, we used Student’s *t-test*. Data shown are averages ± SD.

## Results

### The absence of TOP1α resulted in reduced fertility and impaired the process of meiosis

**Figure 1:**
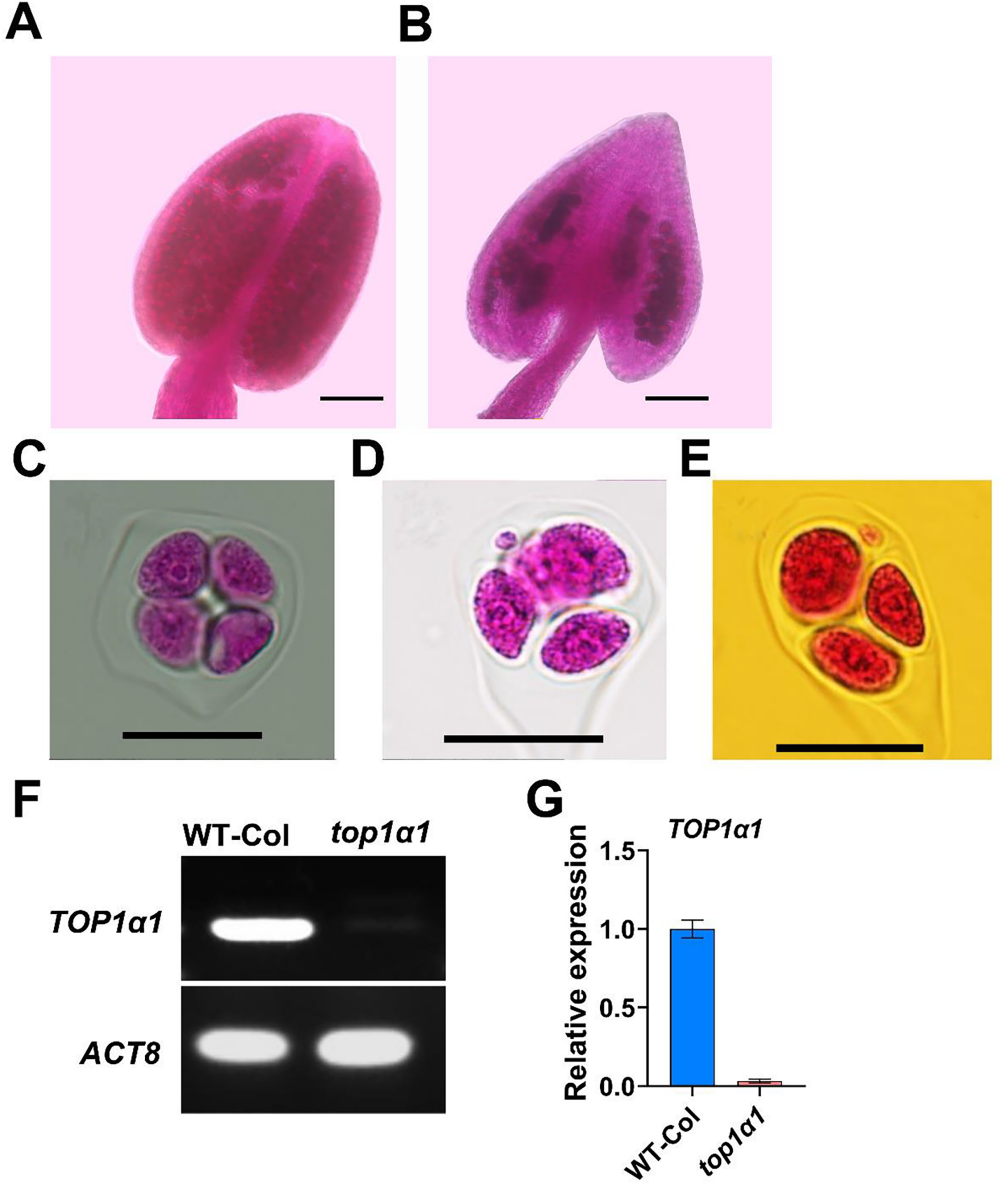
*top1α1* plants exhibited reduced fertility and induced abnormal meiotic products. The anther of the wild-type exhibited a density of pollen grains (A), while the anthers of the top1α1 mutant displayed a significantly lower abundance of pollen grains (B). Unlike the wild-type, *top1α1*, tetrads with four microspores contained polyads with small microspores, suggesting the occurrence of a meiotic abnormality. A total of 50 observations were conducted, revealing that 6% of the observed tetrads had abnormalities. A wild-type of tetrad with four microspores (C). *top1α1* polyads with additional small microspores (D and E). $AtTOP1 \alpha$ mRNA is disrupted across the T-DNA insertion sites in the *top1α1* line (F and G). Scale bars = 50 µm (A and B) and 20 µm (C–E).

To investigate how the absence of functional $AtTOP1 \alpha$ affects pollen viability and anther starch accumulation, we used Alexander Red staining to assess pollen viability and PCR analysis to confirm the genotype of *top1α1* mutants. The homozygous mutants developed normally, but with early flowering and differences in leaf shape and internodal distance (Takahashi et al., 2002). Since the T-DNA insertion disrupts $AtTOP1 \alpha$ gene expression, a continuous mRNA transcript cannot be generated across the insertion site. Therefore, the expression level of AtTOP1α cannot be determined using the presented semi-quantitative PCR (Fig. 1F) and qPCR (Fig. 1G) data. Our results revealed that the wild-type (WT) anthers contained significantly more pollen grains compared to top1α1 anthers (Figs. 1A and 1B, respectively). We also examined the tetrad stages, while the WT tetrads had four microspores (Fig. 1C), tetrads contained polyads with small microspores, suggesting the occurrence of a meiotic abnormality. A total of 50 observations were conducted, revealing that 6% of the observed tetrads had abnormalities (Figs. 1D and 1E). We also investigated pollen fertility using I2/KI staining and observed many immature pollen grains in the mutant; some of the top1α1 pollen grains were larger than wild-type pollen (Fig. S1).

### The disruption of TOP1α influenced the behavior of chromosomes during prophase

**Figure 2:**
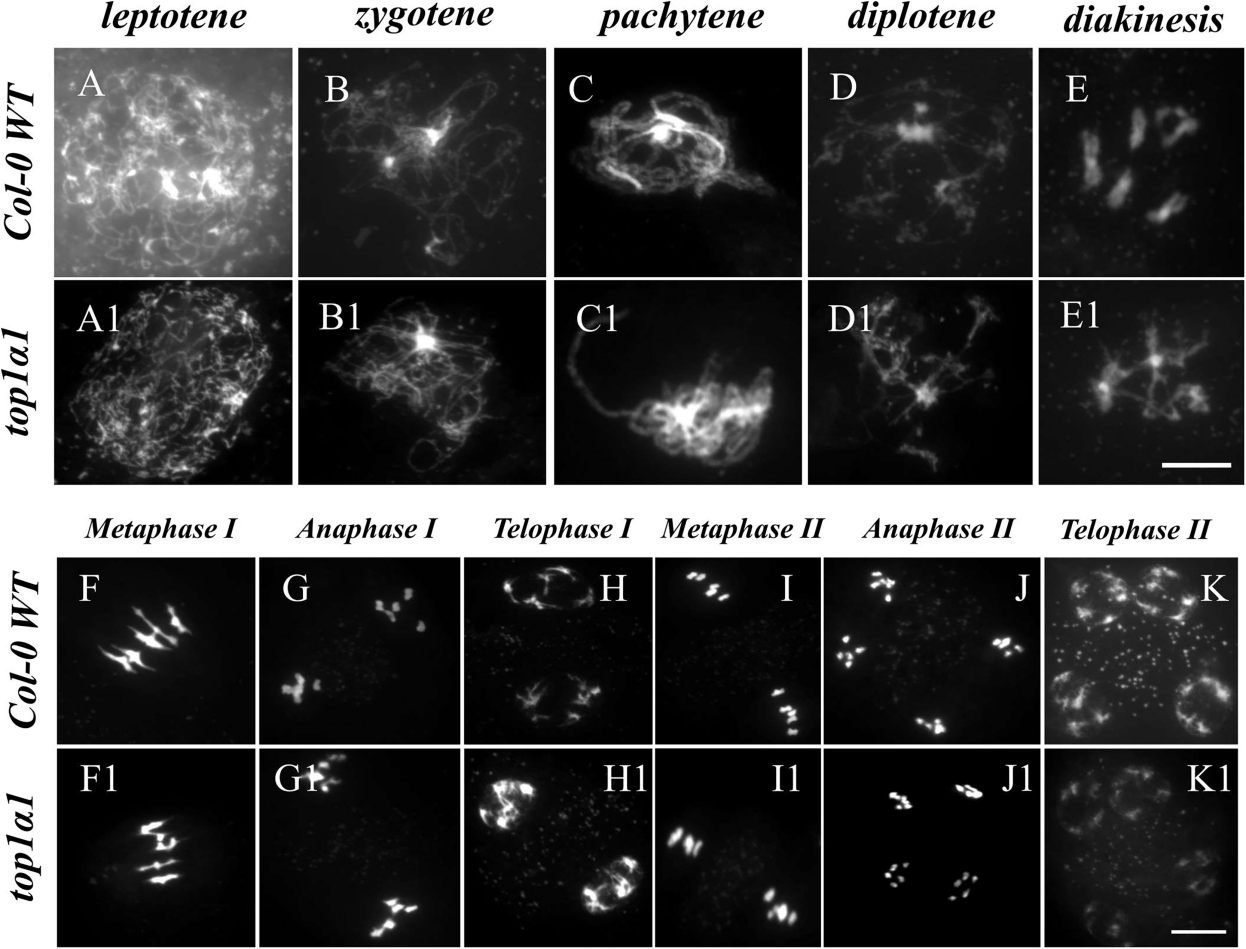
DAPI staining of meiotic chromosomes revealed abnormal chromosomal behavior in top1α1 mutants during prophase I. Wild-type chromosome behavior is shown in (A)-(K), while (A1)-(K1) correspond to top1α1. Unlike the wild-type, top1α1 displayed abnormal chromosome features from zygotene to metaphase I, including the formation of multivalent links between chromosomes instead of normal bivalents and interlocks between homologous chromosomes. Chromosomes were stained with DAPI. Scale bar = 10 µm.

To assess chromosomal behavior during male meiosis in WT and top1α1 mutants, we used DAPI staining. DAPI staining revealed largely normal meiosis in the mutants compared to WT, with minor variations in some stages (Fig. 2). Both WT and *top1α1* meiotic chromosomes condensed at leptotene and displayed generally identical morphologies (Figs. 2A and 2A1, respectively). However, in *top1α1* at zygotene (*n* = 465/589) and pachytene (*n* = 628/807), a chromosome displayed bright large blocks of heterochromatin (Figs. 2B1 and 2C1, respectively). WT meiocytes (*n* = 259) had compact homologs at diplotene (Fig. 2D), but the *top1α1* (*n* = 289/342) chromosomes were fragile, with thinner regions than usual (Fig. 2D1). In diakinesis, the WT (*n* = 196) showed five complete bivalents (Fig. 2E), while some entanglements existed between the bivalents in *top1α1* (*n* = 178/211) (Fig. 2E1). At metaphase I, the WT bivalents (*n* = 192) were regular and lined up (Fig. 2F), while the *top1α1* bivalents (*n* = 99/156) were often thin with a few crossovers and two or more *top1α1* chromosomes frequently linked together (Fig. 2F1). After meiotic II division, chromosome crowding was observed in *top1α1* at anaphase I and telophase I (Figs. 2G1 and 2H1, respectively). The presence of stacking during meiosis I was consistent with the lack of $AtTOP1 \alpha$ function, which plays a critical role in developing appropriate DNA structure and topology, fixing kinks, torsions, and dealing with tight-twisted intermediates produced during replication.

### TOP1α is required to untangle chromosomes at the centromere regions

**Figure 3:**
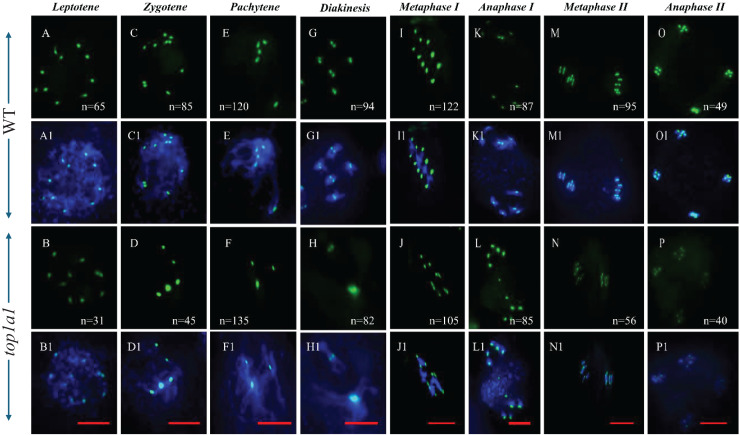
*top1α1* induces unbalanced chromosome segregation. FISH analysis with a centromere probe. (A–P) Wild-type (A, C, E, G, I, K, M, O) and top1α1 (B, D, F, H, J, L, N, P) meiotic cells at leptotene (A and B), zygotene (C and D), pachytene (E and F), diakinesis (G and H), metaphase I (I and J), anaphase I (K and L), metaphase II (M and N), and anaphase II (O and P) stages. FISH with a centromere probe revealed a similar number of signals in wild-type and top1α1 cells at leptotene (10 in wild-type and 8–10 in top1α1). In wild-type cells, the number of centromeric foci decreased from 10 at zygotene to 5 at pachytene. In contrast, top1α1 cells displayed fewer and larger foci at zygotene (4 foci) and pachytene (3 foci). While both wild-type and top1α1 diakinesis and metaphase I cells had the expected five pairs (10 signals) of centromeres, top1α1 cells frequently exhibited univalent and multivalent chromosomes. No significant differences were observed between WT and top1α1 cells from anaphase I to telophase II. Blue: DAPI staining of chromosomes; Green: centromere FISH signals. *n* = number of cells observed with the corresponding phenotype. Scale bar = 10 µm.

The centromere is a region on each linear chromosome essential for ensuring proper chromosome segregation during cell division. To test whether the absence of the TOP1α function affects the disentanglement at the chromosome level during meiosis I, we performed FISH using a centromere probe on top1α1 meiotic cells to examine chromosome pairing and synapsis, to see whether *top1α1* bivalents occurred between homologs or non-homologs. FISH analyses with a centromere probe exhibited a similar number of signals in WT and *top1α1* at leptotene, with wild-type cells displayed 10 centromere foci, while top1α1 cells had 8–10 foci (Figs. 3A and 3B, respectively).

At zygotene and pachytene, Col-0 WT cells had 10 (*n* = 85) and 5 (*n* = 120) centromeric foci, respectively (Figs. 3C and 3E); in contrast, there were fewer and larger foci in *top1α1* at zygotene, ranging between 6 (~16%, 28/175), 5 (~26%, 45/175), 4 (~23%, 40/175), 3 (~23%, 40/175), 2 (~9%, 15/175), and 1 (~3%, 5/175) (Fig. 3D). Several *top1α1* cells at the zygotene stage also displayed clustered foci in one location compared to WT (Fig. S2).

In *top1α1*, the number of chromosome signals decreased at the pachytene stage. The number of foci ranged from 5 (~11.5%, 30/260), 4 (~19%, 50/260), 3 (~52%, 135/260), 2 (~13.5, 35/260), to 1 (~4%, 10/260), with a large signal (Fig. 3F), which was confirmed by multiple cell analysis of *top1α1* at the pachytene stage (Fig. S3).

At diakinesis, the WT had five pairs of homologous chromosomes and five centromeric foci (Fig. 3G). In contrast, the *top1α1* chromosomes were grouped and overlapping, with signals concentrated in two locations (Fig. 3H). At metaphase I, in WT, five pairs of centromere foci on five bivalents were positioned on a single line (Fig. 3I), while in *top1α1* we observed that, chromosomes were in abnormal alignment, and there was a tangle between non-homologous chromosomes but also 10 foci (Fig. 3J), revealing a non-homologous association. Results for WT and *top1α1* cells from anaphase I to telophase II were similar (Fig. 3). From these observations, we suggest that *top1α1* had abnormalities in pairing and synapsis around the centromeres.

In many organisms, telomeres are arranged as “a bouquet” on the nuclear envelope to promote pairing and synapsis. While *Arabidopsis* does not have a traditional bouquet, it is believed that the same outcome is achieved by clumping telomeres around the nucleolus before leptotene. We observed this “clumping” pattern in both WT and top1α1 telomere foci at leptotene (Figs. S4A and S4B, respectively), at zygotene (Figs. S4C and S4D, respectively), and at pachytene (Figs. S4E and S4F, respectively), and we found WT and top1α1 both had similar numbers of signals (9–10, 10 and 10 telomere foci, respectively).

### Disruption of TOP1α affected 45s DNA localization in *A. thaliana*

**Figure 4:**
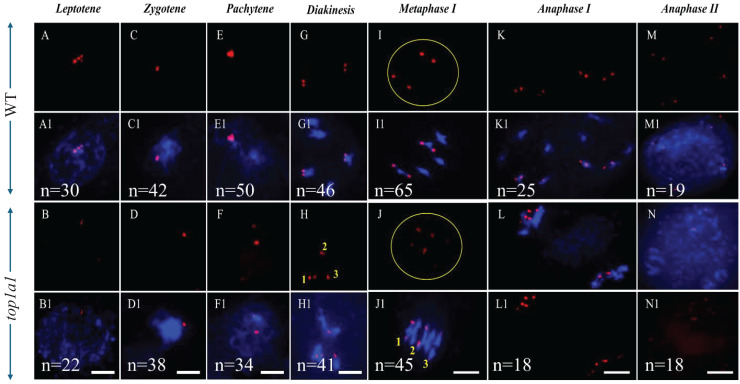
FISH with a 45S rDNA probe revealed the same number of signals in wild-type and *top1α1* plantsat leptotene and zygotene. At pachytene, the WT showed a single intense 45S rDNA signal, while top1α1 displayed two separate signals. At metaphase I, WT signals were located on two bivalent chromosomes (2–4), whereas top1α1 signals were on two bivalents and a univalent chromosome. Both WT and top1α1 cells displayed similar foci at diakinesis, anaphase I, and anaphase II. Blue indicates DAPI-stained chromosomes; red indicates 45S rDNA FISH signals. Scale bar = 10 µm.

We conducted FISH analysis targeting 45S rDNA to examine chromosome pairing in *top1α1* plants compared to WT. The 45s rDNA fociin WT and *top1α1* were identical at leptotene and zygotene (Figs. 4A, 4C and 4B, 4D, respectively). At pachytene, the WT had a single intense signal (Fig. 4E), while the majority of the *top1α1* samples had two separate signals (Fig. 4F), which indicates partial separation of the 45S rDNA regions. This result shows that homologous chromosomes in *top1α1* were incorrectly paired and synapsed easily at the 45S rDNA region. We also observed the presence of signals on three bivalents in *top1α1* at diakinesis and metaphase I (Figs. 4H and 4J, respectively) and more images at metaphase I (Fig. S5), to illustrate these pairing abnormalities in *top1α1* cells at metaphase I.

### TOP1α promotes crossover in *A. thaliana*

**Figure 5:**
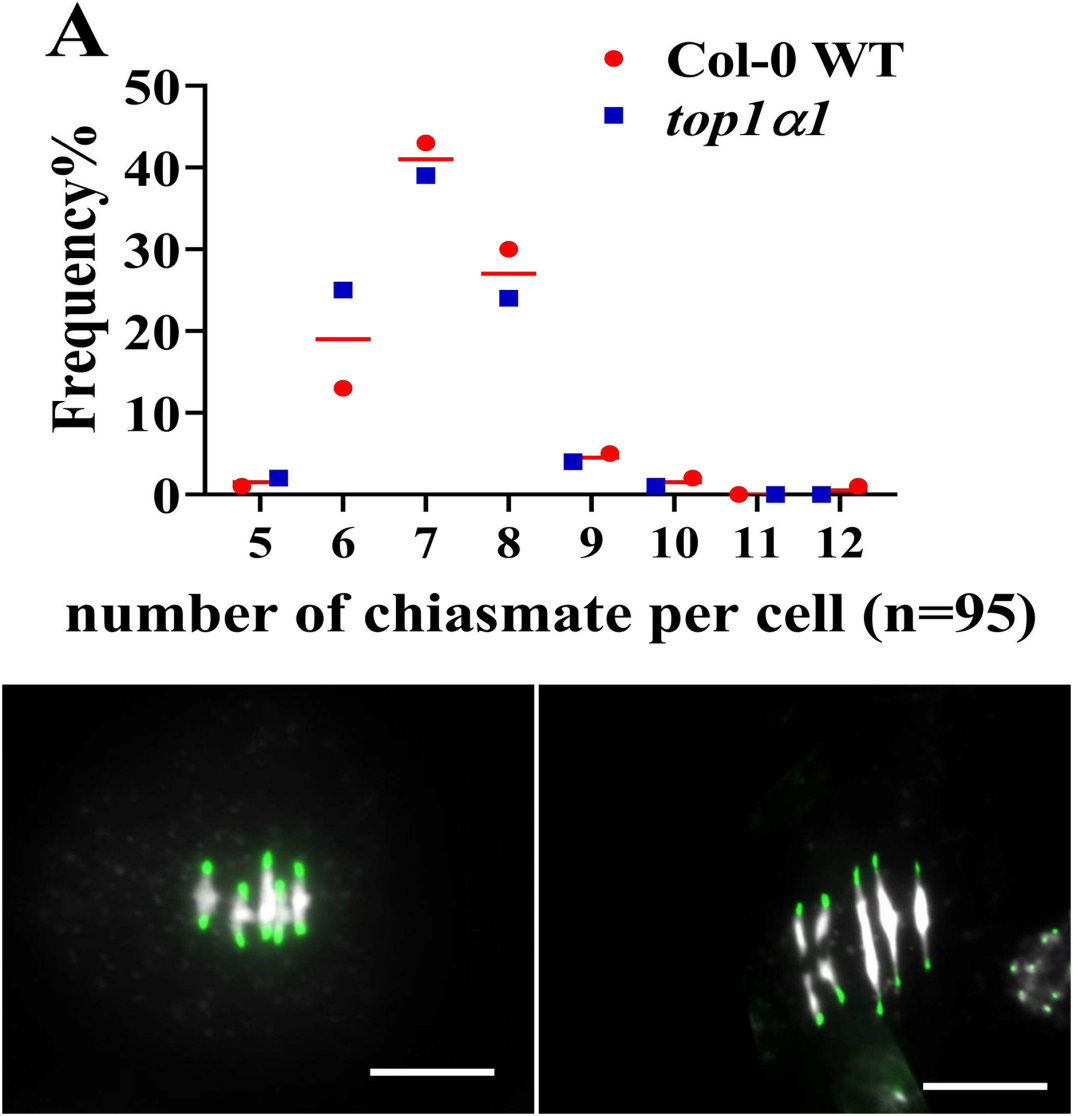
Comparing the average number of chiasmata formations per one PMC in the wild-type and *top1α1* at metaphase I.

Visualizing of chiasma during diplotene and diakinesis in *Arabidopsis thaliana* is hindered by the difficulty in differentiating between true chiasmata and non-recombinant twists of homologous chromosomes. Additionally, the close association of nucleolus organizer regions (NORs) with the nucleolus in the short arms of chromosomes 2 and 4 impedes chiasma identification in these regions. Therefore, despite the condensed state of chromosomes at metaphase I, this stage offers a more suitable platform for chiasma scoring in Arabidopsis. We investigated and documented chiasma frequency in wild-type (WT) and top1α1 mutant pollen mother cells (PMCs) at metaphase I using fluorescence *in situ* hybridization (FISH) labeling with a centromeric probe, following established protocols. Analysis revealed a consistent pattern of five bivalents formed by the five chromosome pairs in all examined cells. These bivalents could be categorized as rods or rings. Rods displayed chiasmata in only one chromosome arm, while rings exhibited chiasmata in both arms. Figure 5 demonstrates a significant difference in chiasma number between WT and top1α1. The mutant exhibited an increased frequency of cells containing 5, 6, or 7 chiasmata compared to WT. Conversely, WT had a higher proportion of cells containing more than seven chiasmata. This suggests that functional TOP1α does not influence crossovers in Arabidopsis. Based on these findings, we conclude that TOP1α promotes crossover events in Arabidopsis.

### TOP1α acts synchronously with ATM during DNA repair

**Figure 6:**
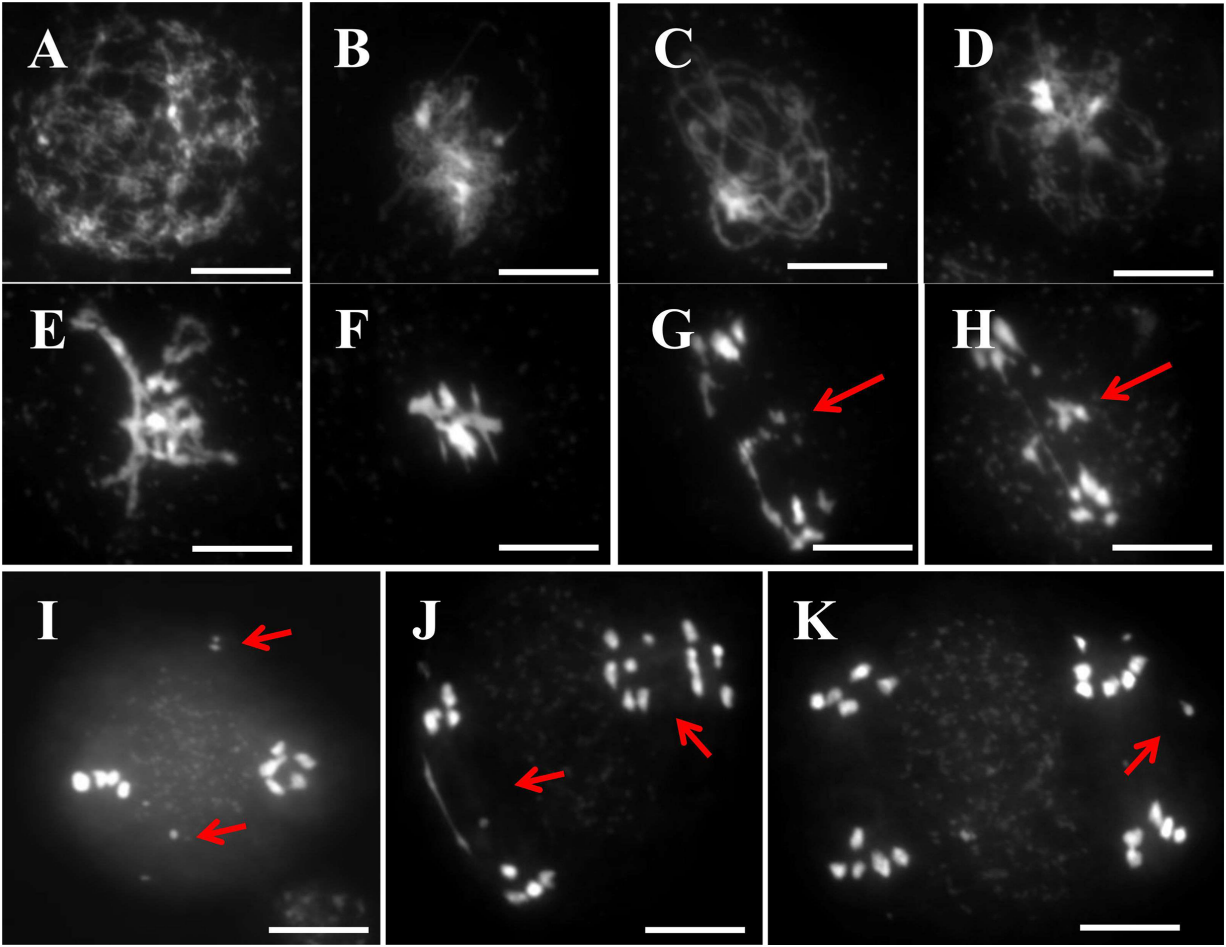
The meiotic chromosomal behavior of atm (by DAPI). (A) Leptotene, (B) zygotene, (C) pachytene. (D) At diplotene, the beginning of the emergence of interlocking and overlapping between non-homologous chromosomes. (E) At diakinesis, intertwined between the non-homologous chromosomes. (F) At metaphase I, atm formed a multivalent link between three or more chromosomes and univalent link. From anaphase I (G and H) through telophase II (K), bridges between chromosome groups and chromosome fragmentation are observed. Telophase II cells also exhibit unequal chromosome numbers. White indicates chromosomes stained with DAPI. Scale bar = 20 µm. Red arrows indicate incomplete migration of chromosomes.

The atm mutant is known to have defects in meiosis and produces fewer numbers of seeds. While the pachytene stage in atm PMCs appears normal, diakinesis shows intertwining between the non-homologous chromosomes (Fig. 6E). At metaphase I, multivalent links involving three or more chromosomes form alongside univalents (Fig. 6F). Bridges between chromosome groups and chromosome fragmentation are observed throughout anaphase I (Figs. 6G and 6H), metaphase II (Fig. 6I), anaphase II (Fig. 6J), and telophase II (Fig. 6K). Telophase II cells also exhibit unequal chromosome numbers (Figs. 6G, 6I, 6J, 6K).

**Figure 7:**
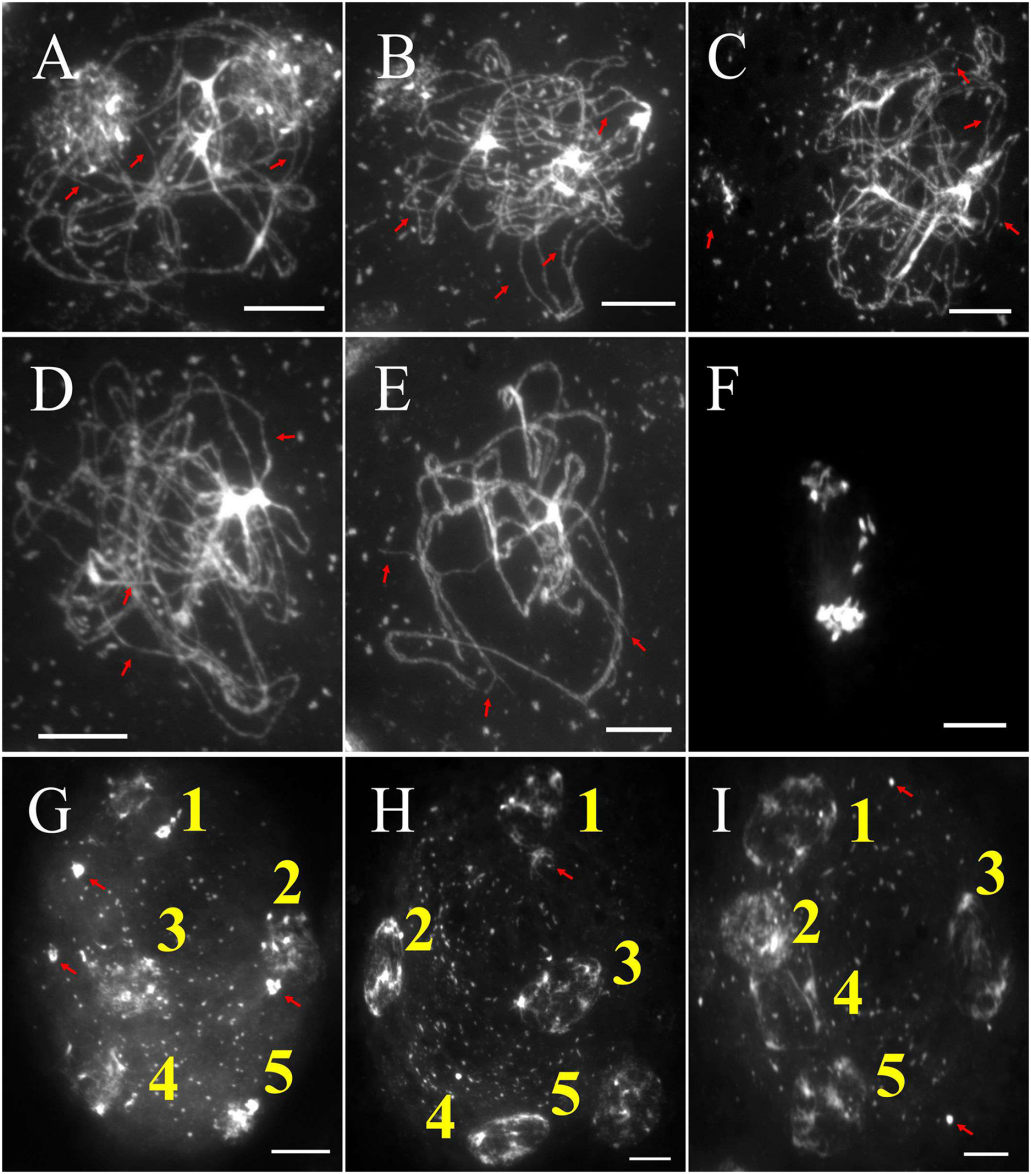
The meiotic chromosomal behavior *atm*×*top1α1* (by DAPI). (A–C) Zygotene, (D and E) pachytene, (D) anaphase I, (G–I) telophase II. White indicates chromosomes stained with DAPI. Scale bar = 10 µm. (A–E) Red arrows indicate incomplete synapsis. (G–I) The red arrows indicate the presence of chromosome pieces that did not migrate to one of the daughter cells.

We developed the *atm×top1α1* double mutant and examined its chromosomal behavior. Compared tothe single-atm mutant, we observed incomplete synapsis of homologous chromosomes at zygotene and pachytene (Figs. 7A–7C and 7D, 7E, respectively). Additionally, some DNA fragments fail to segregate to cell poles at anaphase I (Fig. 7F). These defects lead to the production of multiple polyads (five unbalanced microspores) at telophase II in the double mutant (Figs. 7G–7I).

## Discussion

The defects in meiotic processes can cause genome instability or even death in proliferating cells (Hou et al., 2021). Here, we demonstrate that *Arabidopsis* TOP1α is required for chromosome relaxation during meiosis. Large distances of heterochromatin at early stages (zygotene and pachytene), also chromosome compacted (Figs. 2B1 and 2C1, respectively), were observed in top1α1 mutant. This finding aligns with the established role of TOP1α in relieving torsional stress during DNA processes (Tan & Tse-Dinh, 2024).

In mammalian cells, centromeric chromatin distribution is regulated epigenetically (Morrison & Thakur, 2021). When we performed FISH analysis with a centromere probe, we observed clusters of signals at the heterochromatin sites in zygotene and pachytene (Fig. 3). This was evident in most cells in both zygotene (Fig. S2) and pachytene (Fig. S3). Therefore, we suggest that TOP1α has an essential role in regulating the centromeric region, which controls the regulation of multiple functions.

In addition, a defect in 45s was observed when using a 45s rDNA probe with FISH (Figs. 4 and S5). From pachytene to late stages, the signal was divided into two halves at pachytene in the top1α1 mutant and observed on three bivalents in the top1α1 mutant, instead of two in the WT diakinesis and metaphase I. These findings also suggest that TOP1α plays a role in organizing of centromeric and pericentromeric regions properly. Meiotic centromeres tend to disassemble, but transient proximity to telomeres ensures they reassemble (Klutstein et al., 2015). TOP1α affected the reassembly of the centromeric regions after prior disassembly (Hou et al., 2021; Shao et al., 2021).

Similar to yeast and humans, *Arabidopsis* exhibits at least two functionally distinct classes of crossover (CO) events (France et al., 2021; Singh et al., 2023). Class I CO is sensitive to interference and requires the ZMM proteins Zip1, Zip2, Zip3, Zip 4/Spo22, and Msh4/Msh5 (Dluzewska et al., 2023). In contrast, Class II COs depend on MUS81 and are interference-insensitive (Li et al., 2021). We observed reductions in chiasma frequency in *top1α1* compared with the WT (Fig. 5); this loss of COs is consistent with the region’s instability between the centromere and telomere (de Massy, 2013; Ito & Shinohara, 2023; Kim & Choi, 2022). To examine CO formation in *top1α1*, we applied DAPI staining combined withFISH to metaphase I chromosome spreads to observe chiasmata in chromosome pairs (Armstrong, 2013). The chiasmata count indicated a decrease in the total COs in *top1α1* compared to the WT (Fig. 5). We, therefore, conclude that TOP1α affects crossover occurrence through the regulation of DNA torsion. DNA topoisomerases are critical enzymes responsible for relaxing supercoiled DNA during DNA duplication, transcription, and other cellular transactions by decreasing the strain in DNA. These enzymes enable the conversion between single-stranded and double-stranded DNA (Duprey & Groisman, 2021; Jian & Osheroff, 2023; Soren et al., 2020).

ATM is primarily recognized for its pivotal role in the DNA damage response, functioning as a sentinel for detecting and initiating signaling cascades in response to DSBs. In contrast, the TOP1α role centers on resolving topological challenges that emerge during DNA replication and transcription processes (Zhang et al., 2022b). In various organisms, ATM functions as a negative regulator of meiotic double-strand break (DSB) initiation (Kurzbauer et al., 2021; Láscarez-Lagunas et al., 2022). We suggest that exacerbated defects observed in the double mutant which exhibited incomplete synapsis and chromosome fragmentation compared to the atm-2 single mutant, were due to the absence of the functional TOP1α affecting DNA repair (Fig. 7). The double mutant also exhibited polyad at telophase II (Figs. 7G–7I), demonstrating that TOP1α and ATM play a critical role in DNA repair. Our study sheds light on the intricate roles of ATM and TOP1α in the context of meiotic DNA repair. These two genes, while sharing a common involvement in DNA repair processes, exhibit distinct mechanisms and operate within different cellular pathways.

A notable aspect of our findings is the potential for synergistic interactions between ATM and TOP1α within the context of the double mutant. Although these genes may not be explicitly aligned within the same canonical DNA repair pathway, the cumulative loss of ATM and TOP1α in the double mutant suggests the possibility of a more pronounced defect in meiotic DNA repair. This observation hints at the likelihood of a functional interplay or redundancy between these genes, whereby their combined absence yields a notable impact on the repair machinery.

## Conclusions

This study provides valuable insights into the involvement of DNA topoisomerase 1α (AtTOP1 α) in male reproductive development in *A. thaliana*. The findings demonstrate that $AtTOP1 \alpha$ plays a role in the meiotic division process, as its absence leads to aberrant chromatin behaviors and various defects during meiosis. Specifically, the top1α1 mutant displays notable variation in heterochromatin distances, clusters of centromere signals, defective distribution of 45s rDNA signals, and reduced frequency of chiasma formation. Furthermore, the *atm-2×top1α1* double mutant exhibits additional defects, including incomplete synapsis, DNA fragmentation, and the occurrence of polyads. Moreover, it suggests that $AtTOP1 \alpha$ functions in conjunction with ATM in the same DNA repair pathway. This study contributes to our understanding of the molecular mechanisms underlying male reproductive development and sheds light on potential targets for further research. It is important to acknowledge that the precise nature of this interaction and whether ATM and TOP1α converge on common downstream effectors necessitate further in-depth investigation. While our study provides a foundational exploration of the roles played by these genes in meiotic DNA repair, we recognize the need for more comprehensive mechanistic studies to dissect their individual contributions and uncover any potential crosstalk. These future investigations will be essential for elucidating the exact mechanisms underpinning the observed phenotypes and refining our understanding of the complex interplay between ATM and TOP1α in DNA repair during meiosis. Overall, this study highlights the importance of DNA topoisomerase 1α in male reproductive development and opens up avenues for future research to unravel its precise roles and mechanisms in plant reproduction.

## Supplemental Information

10.7717/peerj.17864/supp-1 Supplemental Information 1 Supplementary tables.

10.7717/peerj.17864/supp-2 Supplemental Information 2 QPCR raw data.

10.7717/peerj.17864/supp-3 Supplemental Information 3 Number of chiasmata formations per one PMC raw data.

10.7717/peerj.17864/supp-4 Supplemental Information 4 Uncropped Gels/Blots.

10.7717/peerj.17864/supp-5 Supplemental Information 5 Decreased pollen viability in *top1α1*. Pollen viability was determined using the I_2_KI staining process. Microscopic analysis revealed large number of pollen grains that did not reach full maturity. Red arrows indicate abnormal pollens and blue arrows indicate normal pollens in top1α1. Scale bar = 50 µm.

10.7717/peerj.17864/supp-6 Supplemental Information 6 TOP1α is essential for chromosome segregation at centromeres during prophase I. This figure demonstrates the variation in centromere signal numbers in the *top1α1* at the zygotene stage. *top1α1*exhibits fewer and larger foci at zygotene, ranging from 6 to 1 focus per cell. (A) 6 foci (~16%, 28/175), (B) 5 foci (~26%, 45/175), (C) 4 foci (~23%, 40/175), (D) 3 foci (~23%, 40/175), (E) 2 foci (~9%, 15/175), (F) 1 focus (~3%, 5/175). White and blue indicate chromosomes stained with DAPI; green indicates the centromere signals of FISH. Scale bar = 20µm.

10.7717/peerj.17864/supp-7 Supplemental Information 7 FISH analysis using a centromere probe reveals variation in centromere signal numbers in the *top1α1*mutant at the pachytene stage. Compared to zygotene, the number of centromere signals in *top1α1* cells decreased at pachytene. The foci range from 5 to 1 focus per cell. (A) 5 foci (~11.5%, 30/260), (B) 4foci(~19%, 50/260), (C) 3 foci (~52%, 135/260), (D) 2 foci (~13.5, 35/260), (E) 1 focus (~4%, 10/260). White and blue indicate chromosomes stained with DAPI; green indicates the centromere signals of FISH. Scale bar = 20 µm.

10.7717/peerj.17864/supp-8 Supplemental Information 8 FISH analysis with a telomere probe r eveals similar telomere signals in wild-type and *top1α1* cells at leptotene, zygotene, and pachytene stages. No significant difference in the number of telomere foci (9-10, 10, and 10, respectively) was observed between wild-type and top1α1 cells (n = 45). (A, C, and E) show wild-type, (B, D, and F) show *top1α1*.Blue indicates chromosomes stained with DAPI; red: indicates the telomere signals of FISH. Scale bar = 10 µm.

10.7717/peerj.17864/supp-9 Supplemental Information 9 FISH analysis with a 45s rDNA reveals localization defects in top1α1 cells at metaphase I. In *top1α1* cells, signals were found on only three bivalents (~90%, 45/50), suggesting a defect in 45s rDNA localization. White and blue indicate chromosomes stained with DAPI; red: indicates the 45s signals of FISH. Scale bar = 10µm.

## Additional Information and Declarations

### Data Availability

The following information was supplied regarding data availability: The raw data is available in the Supplemental Files.
