# Supplementary material for: The role of DNA topoisomerase 1α (AtTOP1α) in regulating arabidopsis meiotic recombination and chromosome segregation
Source: PeerJ. 2024 Aug 28;12:e17864. doi: 10.7717/peerj.17864 (PMC11365474; doi:10.7717/peerj.17864)
Supplement: Supplemental Information 1 [file peerj-12-17864-s001.docx]

**Supplemental Table S1.** List of primer sequences for PCR genotyping of mutants .

| Primer name | Sequences (5′→3′) | Purpose |
| --- | --- | --- |
| *top1α1-LP* | TCCATATCTCACCCGCCTGG | *top1α1*-specific primers for PCR genotyping |
| *top1α1-RP* | GATGATGATGAGGCCGACAC |  |
| *LB* | ACCCCAGTACATTAAAAACGTC | *LB-*specific primers for PCR genotyping T-DNA insertion |
| *Atm-LP* | ATCCATGTGGTTCAGTCTTGC | *Atm-*specific primers for PCR genotyping |
| *Atm-RP* | TTGGTATCCTGCAGAGGAAAG |  |
| *SALK LBb1.3* | ATTTTGCCGATTTCGGAAC | *SALK LBb1.3-*specific primers for T-DNA insertion |
| *TOP1α-qRT-F* | ACCAAACGCGTGGGAGAAGA | *TOP1α*-specific primers for qRT-PCR |
| *TOP1α-qRT-R* | ACGGCGCGAGAATCTGTACT |  |
| *ACTIN8-qRT-F* | TGTGACAATGGTACTGGAATGG | *ACTIN8*-specific primers for qRT-PCR |
| *ACTIN8-qRT-R* | TTGGATTGTGCTTCATCACC |  |

**Supplemental Table S2.** Nucleotide sequences of centromere, telomere and 45s rDNA probes used in FISH analysis.

| Probes | Sequences |
| --- | --- |
| 25SrDNA sequence | CCCGCTGAGTTTAAGCATATCAATAAGCGGAGGAAAAGAAACTAACAAGGATTCCCTTAGTAACGGCGAGCGAACCGGGAAGAGCCCAGCTTGAAAATCGGACGTCTTCGGCGTTCGAATTGTAGTCTGGAGAAGCGTCCTCAGCGACGGACCGGGCCTAAGTTCCCTGGAAAGGGGCGCCAGAGAGGGTGAGAGCCCGTCGTGCCCGGACCCTGTCGCACCACGAGGCGCTGTCTACGAGTCGGGTTGTTTGGGAATGCAGCCCCAATCGGGCGGTAAATTCCGTCCAAGGCTAAATACGGGCGAGAGACCGATAGCGAACAAGTACCGCGAGGTAAAGATGAAAAGGACTTTGAAAAGAGAGTCAAAGAGTGCTTGAAATTGTCGGGAGGGAAGCGGATGGGGGCCGGCGATGCGTCCTGGTCGGATGCGGAACGGAGCAATCCGGTCCGCCGATCGATTCGGGGCGTGGACCGACGCGGATTACGGTGGCGGCCTAAGCCCGGGCTTTTGATACGCTTGTGGAGACGTCGCTGCCGTGATCGTGGTCTGCAGCACGCGCCTAACGGCGTGCCTCGGCATCAGCGTGCTCCGGGCGTCGGCCTGTGGGCTCCCCATTCGACCCGTCTTGAAACACGGACCAAGGAGTCTGACATGTGTGCGAGTCAACGGGTGAGTAAACCCGTAAGGCGCAAGGAAGCTGATTGGCGGGATCCTCGCGGGTGCACCGCCGACCGACCTTGATCTTCTGAGAAGGGTTCGAGTGTGAGCATGCCTGTCGGGACCCGAAAGATGGTGAACTATGCCTGAGCGGGGTAAAGCCAGAGGAAACTCTGGTGGAAGCCCGCAGCGATACTGACGTGCAAATCGTTCGTCTGACTTGGGTATAGGGGCGAAAGACTAATCGAACCATCTAGTAGCTGGTTCCCTCCGAAGTTTCCCTCAGGATAGCTGGAGCTCGGACGCGAGTTCTATCGGGTAAAGCCAATGATTAGAGGCATTGGGGGCGCAACGCCTCGACCTATTCTCAAACTTTAAATAGGTAGGACGTGTCGGCTGCTTTGTTGAGCCGTCACACGGAATCGAGAGCTCCAAGTGGGCCATTTTTGGTAAGCAGAACTGGCGATGCGGGATGAACCGGAAGCCGGGTTACGGTGCCCAACTGCGCGCTAACCTAGAACCCACAAAGGGTGTTGGTCGATTAAGACAGCAGGACGGTGGTCATGGAAGTCGAAATCCGCTAAGGAGTGTGTAACAACTCACCTGCCGAATCAACTAGCCCCGAAAATGGATGGCGCTTAAGCGCGACCTATACCCGGCCGTCGGGGCAAGAGCCAGGCCTCGATGAGTAGGAGGGCGCGGCGGTCGCTGCAAAACCTAGGGCGCGAGGCGCGGAGCGGCCGTCGGTGCAGATCTTGGTGGTAGTAGCAAATATTCAAATGAGAACTTTGAAGGCCGAAGAGGGGAAAGGTTCCATGTGAACGGCACTTGCACATGGGTTAGTCGATCCTAAGAGTCGGGGGAAACCCGTCTGATAGCGCTTAAGCGAACTTCGAAAGGGGATCCGGTTAAAATTCCGGAACCGGGACGTGGCGGTTGACGGCAACGTTAGGGAGTCCGGAGACGTCGGCGGGGGCCTCGGGAAGAGTTATCTTTTCTGTTTAACAGCCTGCCCACCCTGGAAACGGCTCAGCCGGAGGTAGGGTCCAGCGGCTGGAAGAGCACCGCACGTCGCGTGGTGTCCGGTGCGCCCCCGGGCGCCCTTGAAAATCCGGAGGACCGAGTGCCGCTCACGCCCGGTCGTACTCATAACCGCATCAGGTCTCCAAGGTGAACAGCCTCTGGTCGATGGAACAATGTAGGCAAGGGAAGTCGGCAAAATGGATCCGTAACTTCGGGAAAAGGATTGGCTC |
| telomere sequence | TTTAGGGTTTAGGGTTTAGGGTTTAGGGTTTAGGG |
| Centromere sequence | cen180_oligo2  GGTGTA GCC AAA GTC CRT ATG AGT CTT TGK  cen180_oligo5  TCT TAT ACT CAA TCA TAC ACA TGA CAT CW  cen180_oligo6  AGT CAT ATT YGA CTC CAA AAC ACT AAC C |
