# Supplementary figures and images for: The role of DNA topoisomerase 1α (AtTOP1α) in regulating arabidopsis meiotic recombination and chromosome segregation
Source: PeerJ. 2024 Aug 28;12:e17864. doi: 10.7717/peerj.17864 (PMC11365474; doi:10.7717/peerj.17864)

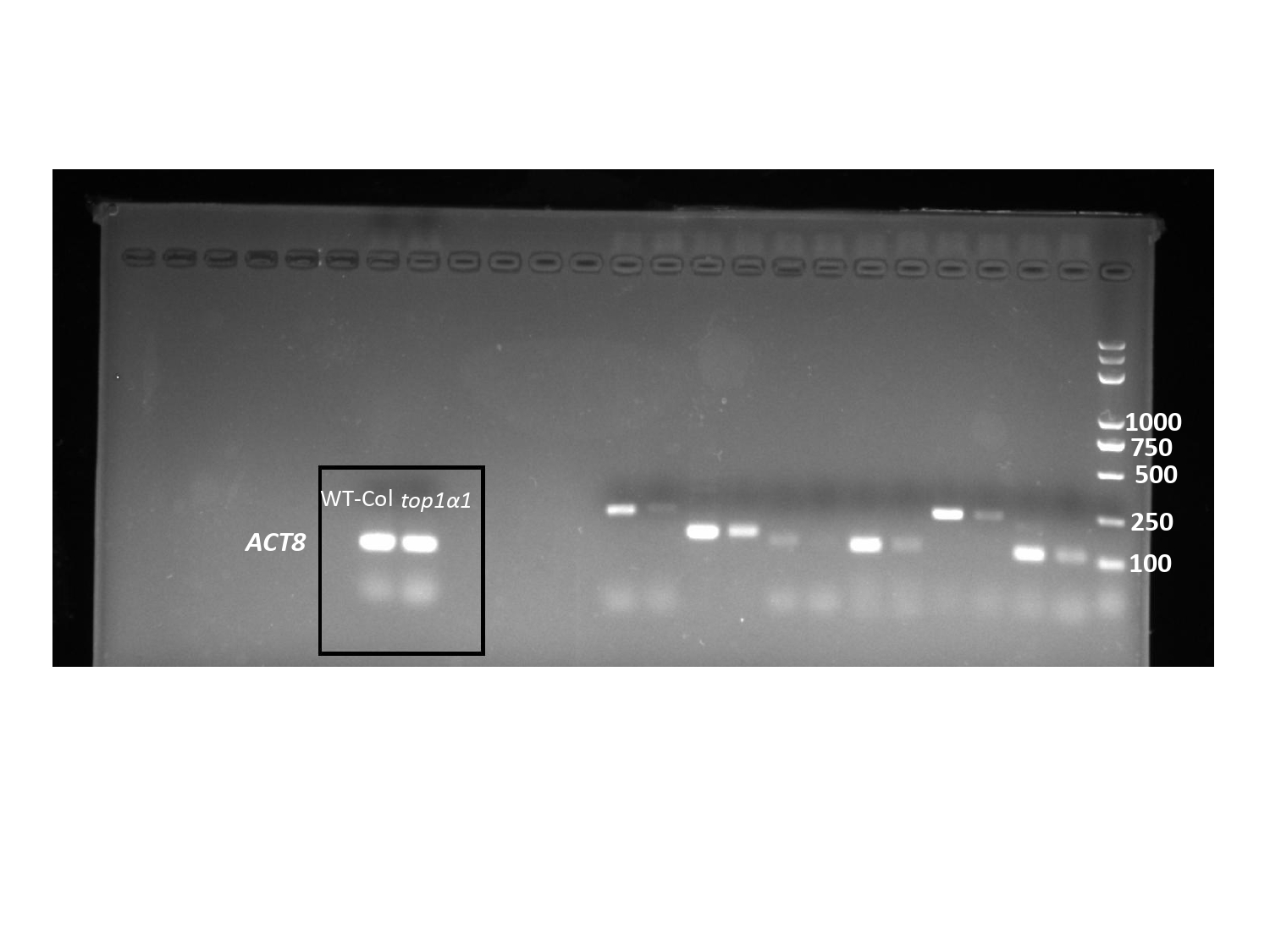

Supplement: Supplemental Information 4 [file peerj-12-17864-s004.zip › Original_page-0001.jpg]

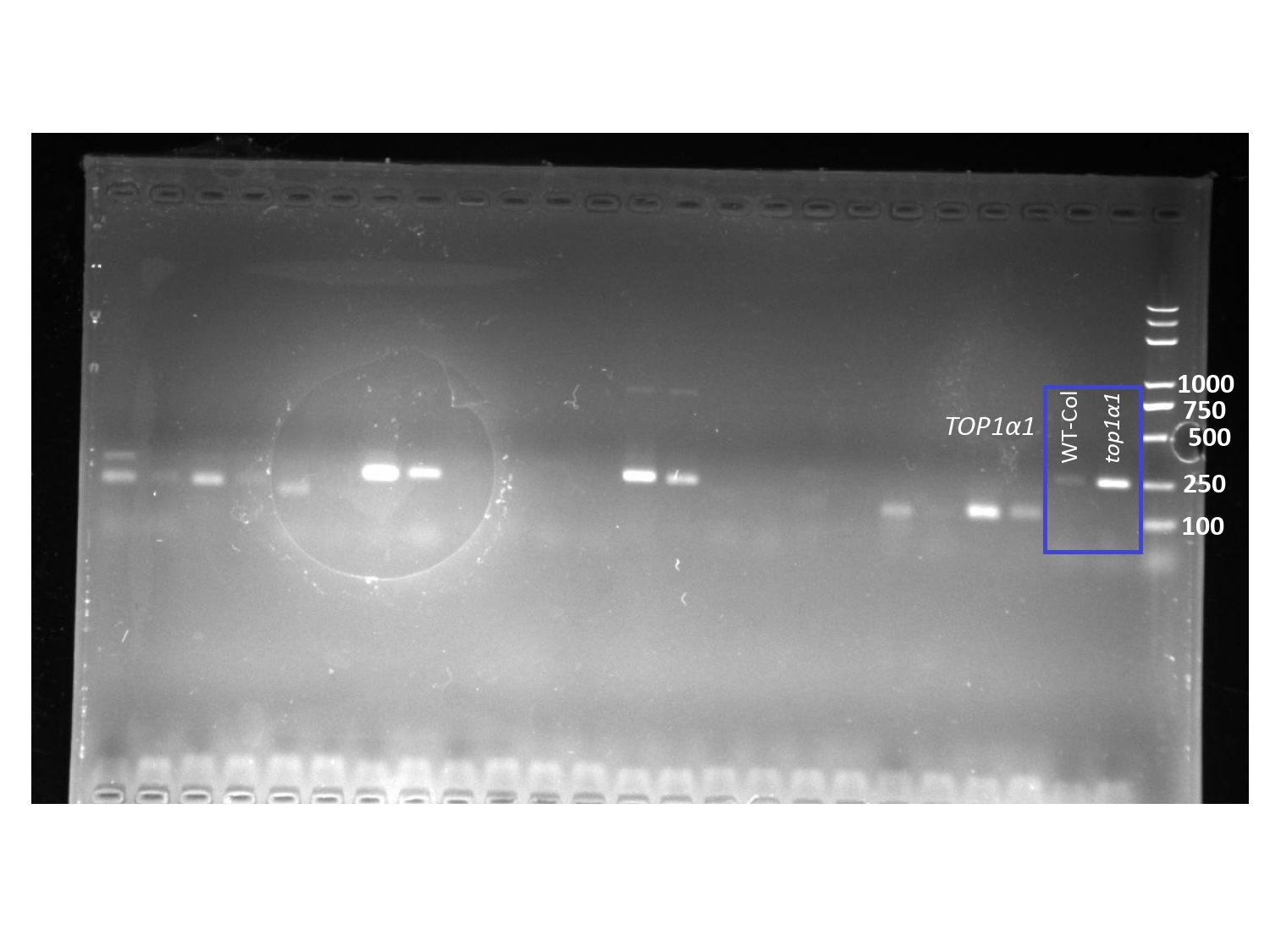

Supplement: Supplemental Information 4 [file peerj-12-17864-s004.zip › Original_page-0002.jpg]

*Col-0 WT*

*top1α1*

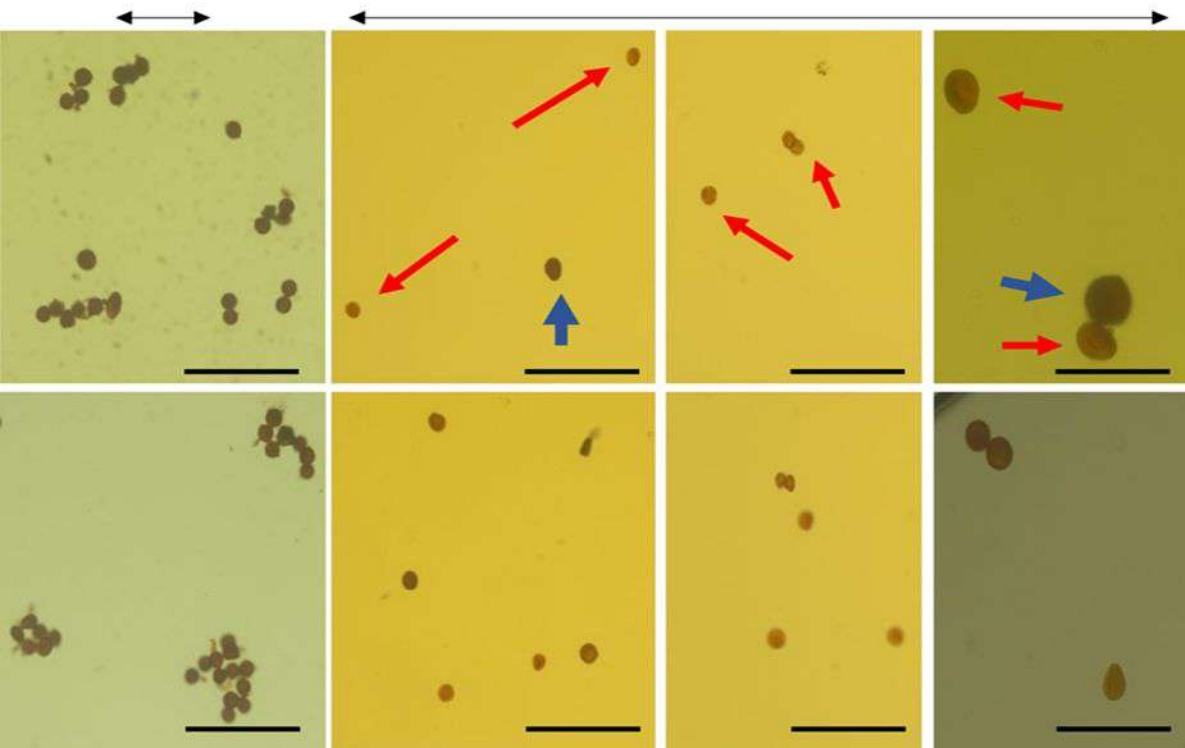

Supplement: Supplemental Information 5 — Pollen viability was determined using the I2KI staining process. Microscopic analysis revealed large number of pollen grains that did not reach full maturity. Red arrows indicate abnormal pollens and blue arrows indicate normal pollens in top1α1. Scale bar = 50 µm. [file peerj-12-17864-s005.pdf]

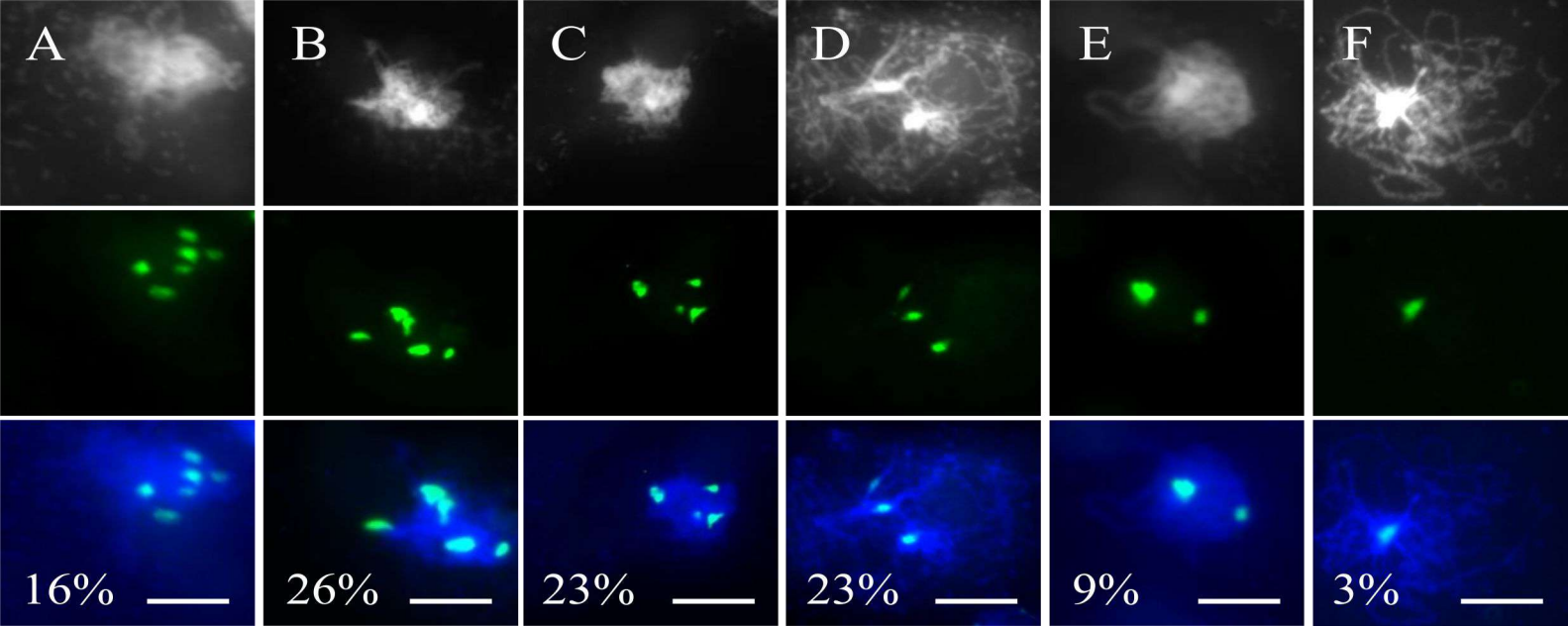

Supplement: Supplemental Information 6 — This figure demonstrates the variation in centromere signal numbers in the top1α1 at the zygotene stage. top1α1exhibits fewer and larger foci at zygotene, ranging from 6 to 1 focus per cell. (A) 6 foci (~16%, 28/175), (B) 5 foci (~26%, 45/175), (C) 4 foci (~23%, 40/175), (D) 3 foci (~23%, 40/175), (E) 2 foci (~9%, 15/175), (F) 1 focus (~3%, 5/175). White and blue indicate chromosomes stained with DAPI; green indicates the centromere signals of FISH. Scale bar = 20µm. [file peerj-12-17864-s006.pdf]

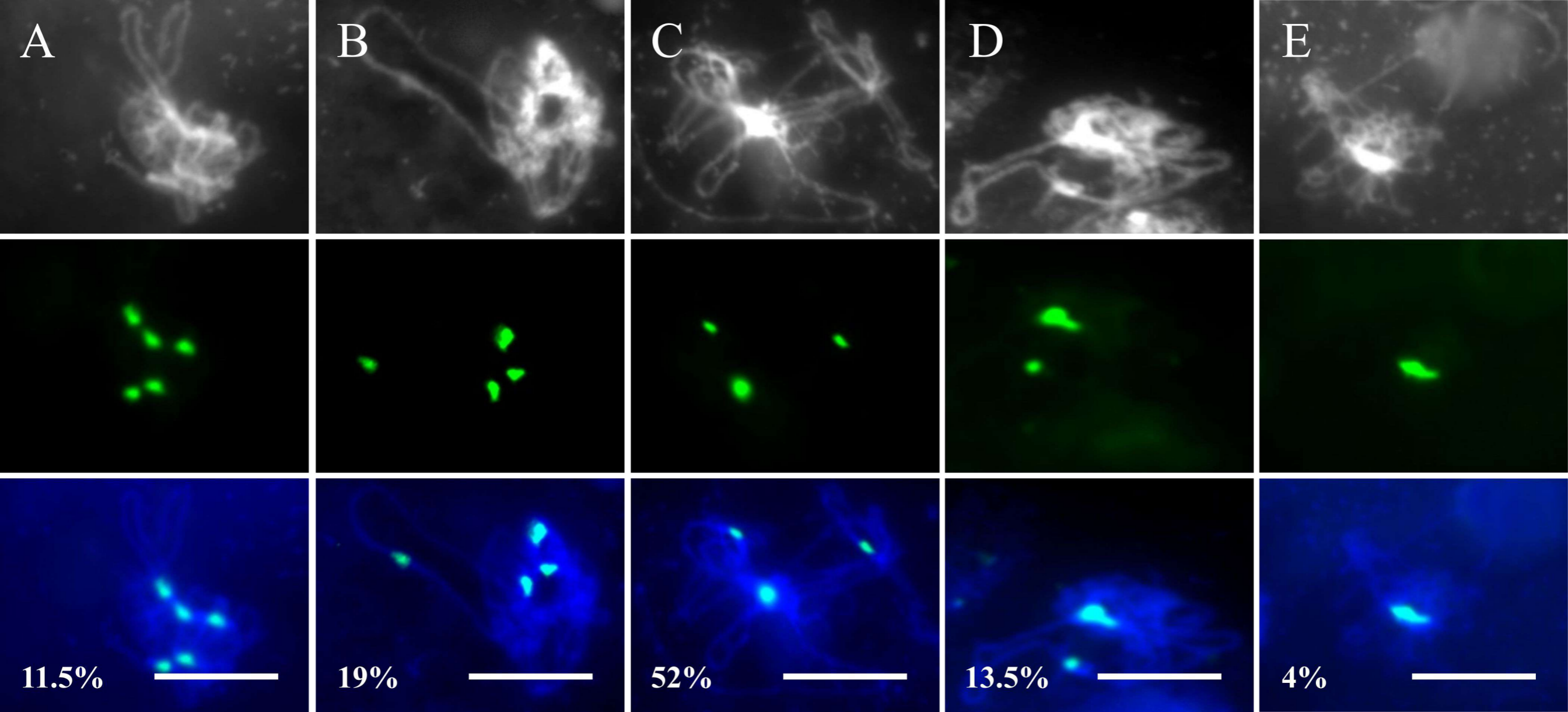

Supplement: Supplemental Information 7 — Compared to zygotene, the number of centromere signals in top1α1 cells decreased at pachytene. The foci range from 5 to 1 focus per cell. (A) 5 foci (~11.5%, 30/260), (B) 4foci(~19%, 50/260), (C) 3 foci (~52%, 135/260), (D) 2 foci (~13.5, 35/260), (E) 1 focus (~4%, 10/260). White and blue indicate chromosomes stained with DAPI; green indicates the centromere signals of FISH. Scale bar = 20 µm. [file peerj-12-17864-s007.pdf]

*Leptotene*

*zygotene*

*pachytene*

A

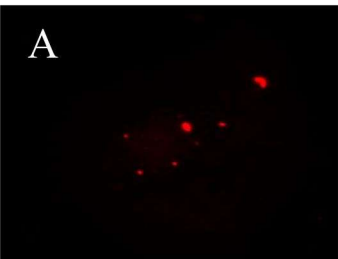

C

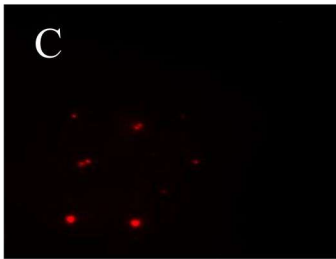

E

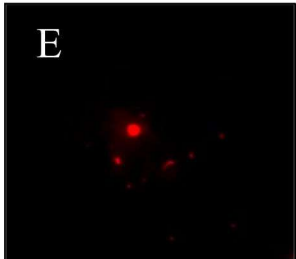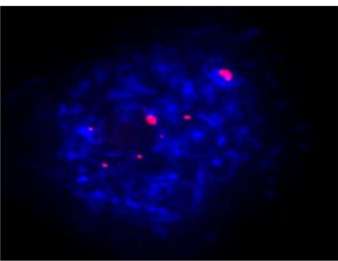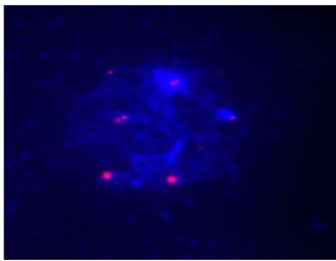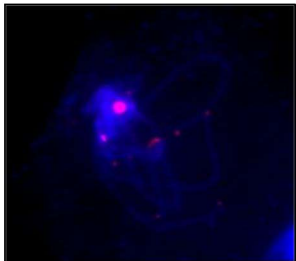

B

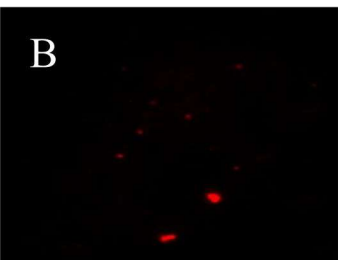

D

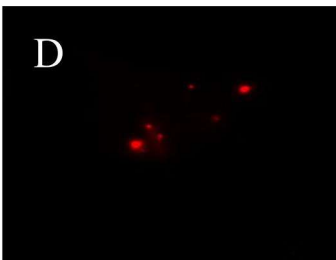

F

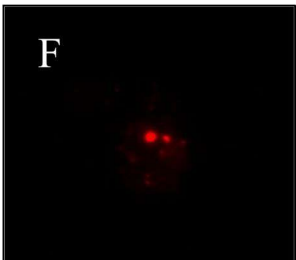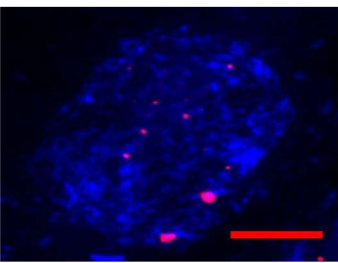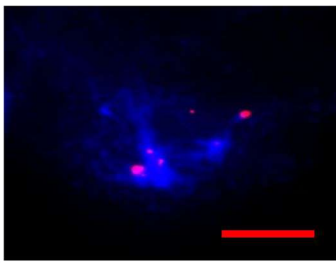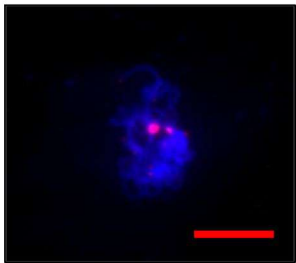

Supplement: Supplemental Information 8 — No significant difference in the number of telomere foci (9-10, 10, and 10, respectively) was observed between wild-type and top1α1 cells (n = 45). (A, C, and E) show wild-type, (B, D, and F) show top1α1.Blue indicates chromosomes stained with DAPI; red: indicates the telomere signals of FISH. Scale bar = 10 µm. [file peerj-12-17864-s008.pdf]

# Metaphase I

*top1 $\alpha$ 1 45s rDNA probe*

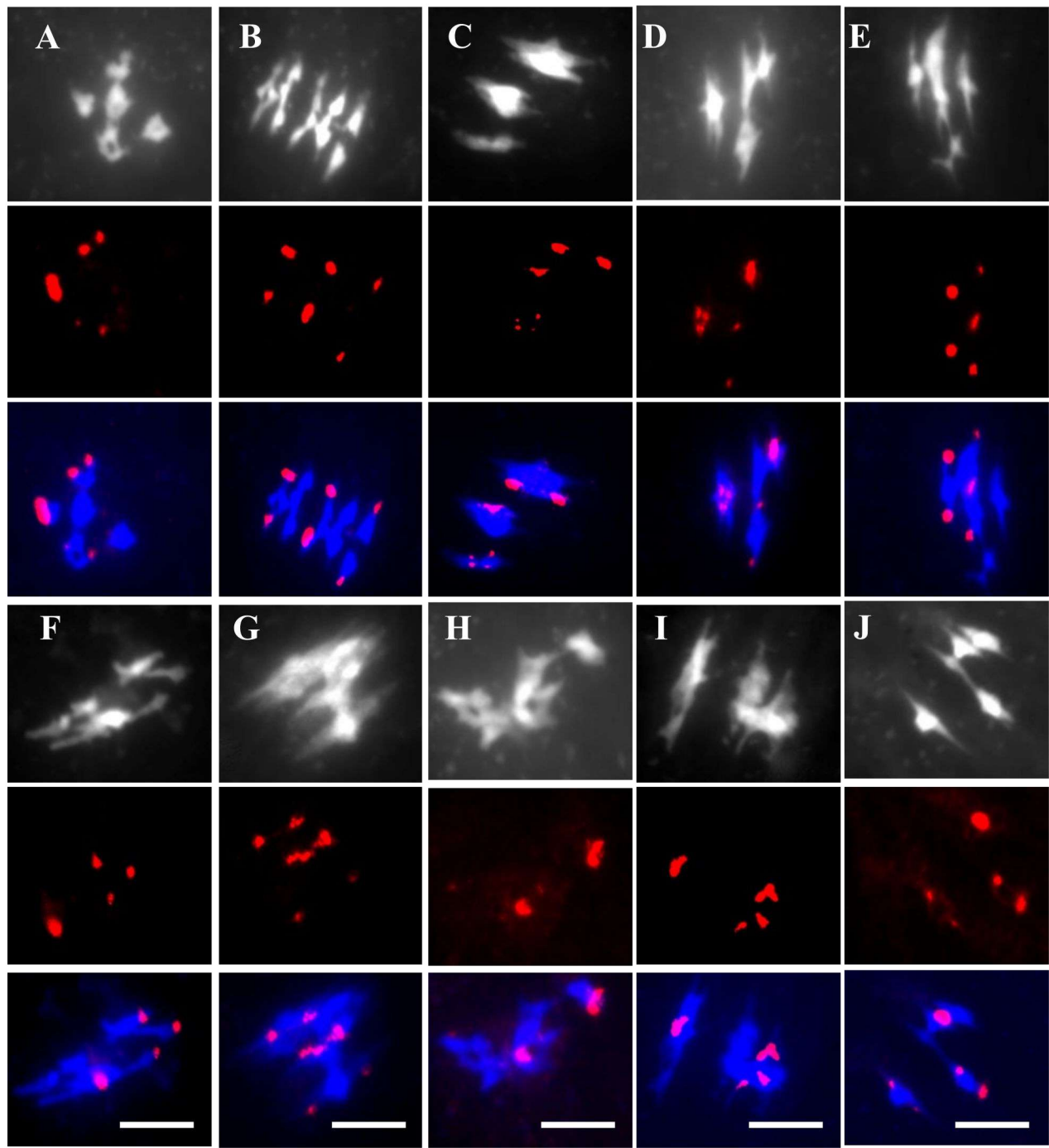

Supplement: Supplemental Information 9 — In top1α1 cells, signals were found on only three bivalents (~90%, 45/50), suggesting a defect in 45s rDNA localization. White and blue indicate chromosomes stained with DAPI; red: indicates the 45s signals of FISH. Scale bar = 10µm. [file peerj-12-17864-s009.pdf]
